# Inhibition of EHMT2/G9a epigenetically increases the transcription of *Beclin-1* via an increase in ROS and activation of NF-κB

**DOI:** 10.18632/oncotarget.9290

**Published:** 2016-05-11

**Authors:** Sang Eun Park, Hye Jin Yi, Nayoung Suh, Yun-Yong Park, Jae-Young Koh, Seong-Yun Jeong, Dong-Hyung Cho, Choung-Soo Kim, Jung Jin Hwang

**Affiliations:** ^1^ Institute for Innovative Cancer Research, Asan Medical Center, Seoul, Korea; ^2^ Asan Institute for Life Sciences, Asan Medical Center, Seoul, Korea; ^3^ Department of Neurology, Asan Medical Center, Seoul, Korea; ^4^ Department of Urology, Asan Medical Center, Seoul, Korea; ^5^ Department of Convergence Medicine, University of Ulsan, College of Medicine, Seoul, Korea; ^6^ Neural Injury Research Lab, University of Ulsan, College of Medicine, Seoul, Korea; ^7^ Department of Urology, University of Ulsan, College of Medicine, Seoul, Korea; ^8^ Graduate School of East-West Medical Science, Kyung Hee University, Yongin, Korea; ^9^ Department of Medicine Engineering, Soon Chun Hyang University, College of Medical Sciences, Asan, Korea

**Keywords:** EHMT2/G9a, histone methyltransferase, Beclin-1, autophagy, epigenetic regulation

## Abstract

We previously reported that BIX-01294 (BIX), a small molecular inhibitor of euchromatic histone-lysine N-methyltransferase 2 (EHMT2/G9a), induces reactive oxygen species (ROS)-dependent autophagy in MCF-7 cells. Herein, we analyzed the epigenetic mechanism that regulates the transcription of *Beclin-1*, a tumor suppressor and an autophagy-related gene (ATG). Inhibition of EHMT2 reduced dimethylation of lysine 9 on histone H3 (H3K9me2) and dissociated EHMT2 and H3K9me2 from the promoter of *Beclin-1.* To this promoter, RNA polymerase II and nuclear factor kappa B (NF-κB) were recruited in a ROS-dependent manner, resulting in transcriptional activation. Moreover, treatment with BIX reversed the suppression of Beclin-1 by the cooperative action of EHMT2 and DNA methyltransferase 1 (DNMT1). Accordingly, a combination treatment with BIX and 5-Aza-2′-deoxycytidine (5-Aza-Cd), a DNMT1 inhibitor, exerted a synergistic effect on Beclin-1 expression. Importantly, high levels of EHMT2 expression showed a significant association with low levels of Beclin-1 expression, which was related to a poor prognosis. These findings suggest that EHMT2 can directly repress Beclin-1 and that the inhibition of EHMT2 may be a useful therapeutic approach for cancer prevention by activating autophagy.

## INTRODUCTION

Autophagy contributes to tumor suppression by removing damaged organelles and misfolded proteins, whereby it acts as a guardian of the genome [1, 2]. Moreover, deficiencies in autophagy have been implicated in both cancer and aging [3–5]. The strongest genetic evidence linking autophagy to tumorigenesis is the emerging supposition that genes that positively control autophagy, such as *Beclin-1,* death-associated protein kinase (*DAPK*), and phosphatase and tensin homolog (*PTEN*), exhibit tumor suppressive functions when assessed in human tumors or cancer models [6–9]. Mammalian *Beclin-1*, an ortholog of the yeast autophagy-related gene6 (*Atg6/Vps30*)—a regulator of the initiation of autophagy, is the first tumor-suppressor gene identified among autophagy-related proteins in human cancer. A high incidence of monoallelic deletion and down-regulation *Beclin-1* have been observed in many breast cancer cell lines and in more than 50% of breast tumors [10]; these phenomena are also common in ovarian and prostate cancers [11–13].

Euchromatic histone-lysine N-methyltransferase 2 (EHMT2/G9a) is a major methyltransferase that catalyzes the mono- and di-methylation of histone H3 lysine 9 (H3K9me and H3K9me2) in euchromatin [14, 15]. EHMT2 plays an important role in gene silencing by associating with DNA methyltransferase (DNMT) and transcriptional repressors during early embryonic development [16]. Recently, abnormally elevated levels of EHMT2 and H3K9me2 have been observed in many types of human cancers. Cancers with these elevated expression levels exhibit silencing of critical tumor suppressor genes or epithelial-to-mesenchymal transition (EMT)-related genes, such as *E-cadherin* [17], *p21* [18], *p14ARF* [19], human mutL homolog 1 (*hMLH1*) [20, 21], runt-related transcription factor 3 (*RUNX3*) [22, 23], desmocollin 3 *(DSC3)*, mammary serine protease inhibitor (*MASPIN*) [24, 25], and *E2F1* [26–28]. The inhibition of EHMT2 has been reported to activate autophagy genes, leading to the transcriptional activation of microtubule-associated protein 1 light chain 3 (*MAP1LC3/LC3*), WD repeat domain phosphoinositide-interacting protein 1 (*WIPI1*), and the diabetes- and obesity-regulated gene (*DOR*) in a c-Jun-dependent manner [29], as well as the expression of B-cell lymphoma 19-kDa interacting protein (*BNIP*) [30]. We previously reported that inhibiting EHMT2 activates autophagy and increases the expression of LC3 and Beclin-1 in MCF-7 cells [31]. Nevertheless, the epigenetic regulation of *Beclin-1* has not yet been elucidated in detail.

Herein, we characterize the epigenetic regulation of *Beclin-1* by EHMT2 and nuclear factor-kappa B (NF-κB) and suggest that it exhibits a relationship with tumorigenesis and patient prognosis in breast cancer.

## RESULTS

### EHMT2 inhibition by BIX induces *Beclin-1* expression in MCF-7 breast cancer cells

**Figure 1:**
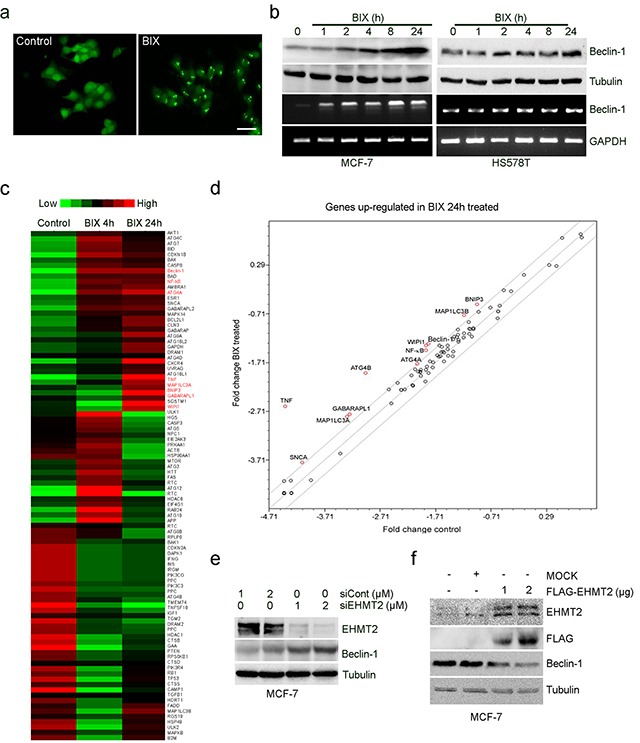
Inhibition of EHMT2 increases Beclin-1 expression **a.** GFP-MCF-7 cells were treated with 10 μM BIX for 2 h. Cells were examined using fluorescence microscopy. Scale bar, 50 μm. **b.** Two breast cancer cell lines (MCF-7, *Beclin-1^+/−^*; HS578T, *Beclin-1^+/+^*) were treated with 10 μM BIX for the indicated amounts of time. Beclin-1 transcripts and protein expression were determined by RT-PCR (lower panel) and Western blotting (upper panel), respectively. **c.** RT2 Profiler PCR analysis for ATG expression. This figure includes a “heat map,” which is the part of the figure containing colors (red, green and black), in which the color represents the expression level of the gene; red and green represent high and low expression, respectively. Expression levels are continuously mapped on the color scale provided at the top of the figure. The heat map represents the differential gene expression pattern in MCF-7 cells treated without or with 10 μM BIX for 4 or 24 h. **d.** Graph represents the fold-change in the level of mRNA transcript in cells treated with BIX for 24 h versus untreated cells. Each circle represents an autophagy gene; those with the greatest fold-changes are indicated in red. The central line represents no changes in expression; above the central line indicates genes with increased expression; below the line are those genes with reduced levels. Grey lines indicate a 2-fold increase or decrease. **e.** MCF-7 cells were transfected with siCont or EHMT2 siRNA (siEHMT2) for 48 h. Western blot analysis was performed using the indicated antibodies. **f.** MCF-7 cells were transfected with 2 μg pcDNA3 empty vector (MOCK) or 1 or 2 μg pcDNA3-Flag-EHMT2 (FLAG-EHMT2) for 24 h; pcDNA3 empty vector was used as a negative control. Western blot analyses were performed using the indicated antibodies.

Using fluorescent microscopy, we could identify LC3-positive puncta in MCF-7 cells that expressed GFP-LC3 after exposure to 10 μM BIX for 4 h (Figure 1a). BIX augmented the levels of endogenous Beclin-1 in a time-dependent manner in the MCF-7 (*Beclin-1^+^*^/−^) and HS578T (*Beclin-1*^+/+^) breast cancer cell lines (Figure 1b). We also assessed the expression of autophagy-related genes using the Human Autophagy RT2 Profiler PCR Array that contained 84 ATGs. Unsupervised clustering analysis revealed distinct patterns of gene expression in MCF-7 cells that were treated with BIX for 4 or 24 h (Figure 1c). The genes identified that showed significant changes in expression (>2-fold) after treatment with BIX were *TNF*, *ATG4*, *WIPI1*, *Beclin-1*, *GABARLAPL1*, *MAP1LC3*, *BNIP3*, and *NF-kB* (Figure 1d and Supplementary Figure S1a–b). Moreover, knockdown of *EHMT2* expression with siRNA also increased Beclin-1 expression (Figure 1e), and the ectopic overexpression of EHMT2 significantly suppressed Beclin-1 expression in MCF-7 cells (Figure 1f). Collectively, these findings suggest that autophagy induced by EHMT2 inhibition was coupled with the transcription of *Beclin-1*.

### Inhibition of EHMT2 activates the transcription of *Beclin-1* in MCF-7 and HS578T cells

**Figure 2:**
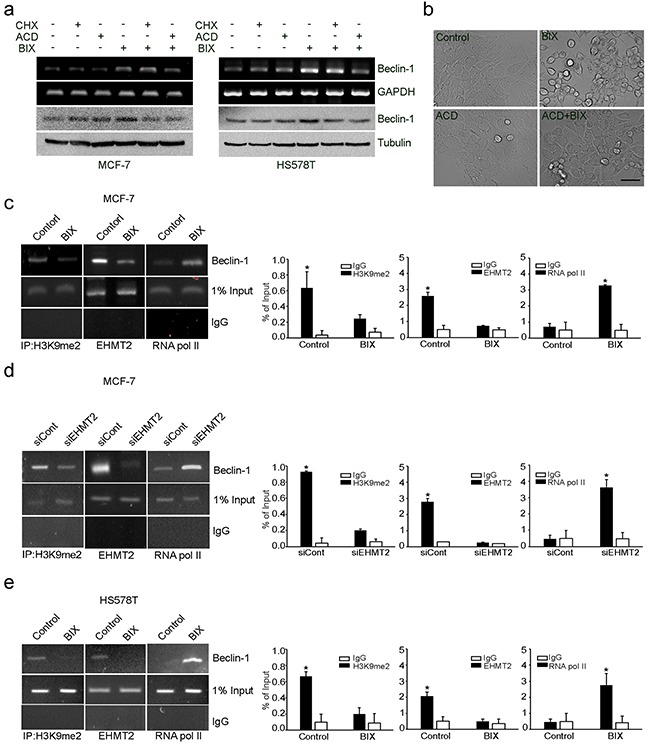
Epigenetic transcriptional activation of *Beclin-1* by EHMT2 inhibition **a.** A pair of breast cancer cell lines (MCF-7: *Beclin-1^+/−^* and HS578T: *Beclin-1^+/+^*) were treated with 10 μM BIX, 10 μg/ml CHX, 1 μg/ml ACD, or combinations of these compounds as indicated for 4 h. Beclin-1 transcripts and protein expression were determined by RT-PCR (upper panel) and Western blotting (lower panel), respectively. **b.** MCF-7 cells were treated with 10 μM BIX and/or 1 μg/ml ACD for 4 h. Cell morphology was examined under a light microscope. Scale bar: 50 μm. **c, d** and **e.** MCF-7 cells and HS578T cells were treated with 10 μM BIX for 4 h or MCF-7 cells were transfected with 1 μM siEHMT2. Treated cells were analyzed by ChIP. The ChIP analysis performed with anti-H3K9me2, anti-EHMT2, and anti-RNA pol II antibodies was compared with normal rabbit IgG as a negative control. An equal amount (input) of DNA–protein complex was applied. Real-time quantification of the *Beclin-1* promoter sequences was carried out with anti-H3K9me2 ChIP, anti-EHMT2 ChIP, and anti-RNA pol II ChIP (right panel). Results are presented relative to the input and are the fold-changes over the control expressed as means ± SEM of three independent experiments. *P < 0.001 compared with control or siCont by one-way ANOVA.

Considering the role of EHMT2 in the transcriptional gene control for autophagy induction, we treated MCF-7 and HS578T cells with BIX in the absence or presence of a translational inhibitor, cycloheximide (CHX), or an RNA synthesis inhibitor, actinomycin D (ACD). CHX reduced the levels of Beclin-1 protein, while ACD reduced the levels of both Beclin-1 mRNA and protein in MCF-7 and HS578T cells exposed to BIX (Figure 2a). Moreover, ACD reduced the characteristic vacuolar phenotype induced by treatment with BIX in MCF-7 cells (Figure 2b). Together, these findings suggested that EHMT2 may control the transcription of *Beclin-1* via an epigenetic mechanism.

To characterize the EHMT2-dependent epigenetic regulation of *Beclin-1*, we mapped its corresponding promoter inside the first intron near the transcriptional start site using chromatin immunoprecipitation (ChIP). We found that the binding of H3K9me2 and EHMT2 to the *Beclin-1* promoter (from 83 to +400) was reduced, whereas the interaction of RNA polymerase II was increased by *BIX* and *EHMT2* siRNA treatment in MCF-7 cells (Figure 2c and 2d). Similar results were obtained in HS578T cells treated with BIX (Figure 2e). These data suggested that increased amounts of H3K9me2 induced by EHMT2 repressed the expression of *Beclin-1* in breast cancer cells.

### NF-κB activates *Beclin-1* transcription in a ROS-dependent manner by BIX

**Figure 3:**
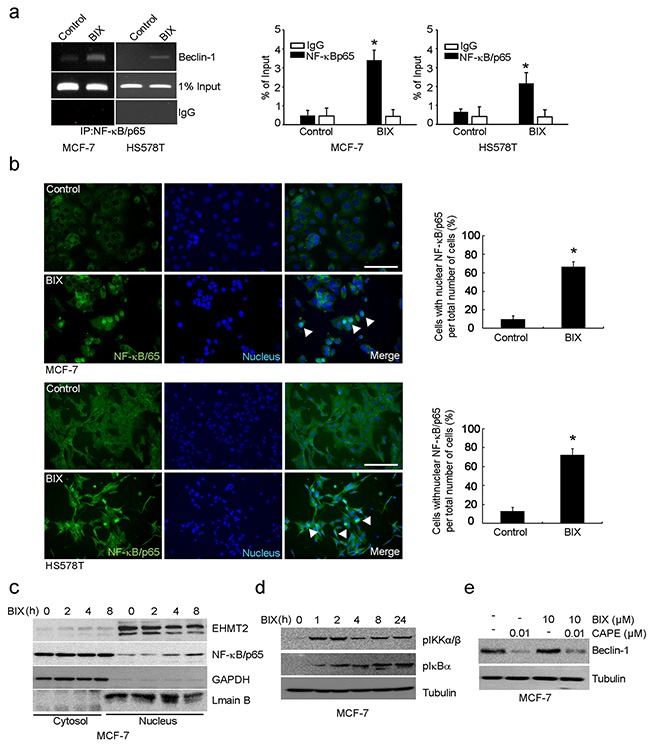
Transcription of *Beclin-1* driven by NF-κB **a.** The ChIP assay reveals the binding of endogenous NF-κB to the *Beclin-1* promoter. Non-specific rabbit IgG was used as an antibody control. MCF-7 and HS578T cells were treated with 10 μM BIX for 4 h and then analyzed. An equal amount (input) of DNA–protein complexes was applied. Real-time quantification of *Beclin-1* promoter sequences was carried out using anti-NF-κB/p65 ChIP in MCF-7 and HS578T cells (right panel). Data are presented relative to the input and are fold-changes over the control value expressed as means ± SEM of three independent experiments. *P < 0.001 compared with the control by one-way ANOVA. **b.** NF-κB was detected using an Alexa488 (green)-conjugated antibody against the 65kDa subunit. Nuclei were visualized with Hoechst 33258 (blue). In control cells, NF-κB/p65 immunoreactivity was preferentially observed in the cytoplasm. At 4 h after the addition of 10 μM BIX, NF-κB/p65 was mostly localized to the nucleus. Scale bar, 50 μm. Right panels show a quantification of NF-κB/p65 translocation. The percentage of cells with nuclear NF-κB/p65 is indicated, and error bars represent the S.D. (four experiments with at least 50 cells analyzed per condition and experiment). Data were analyzed by analySIS TS Auto (mean ± S.D., n = 3; paired *t*-test, *P < 0.001 compared with BIX). **c.** Western blot of BIX induced the nuclear translocation of NF-κB/p65. Nuclear and cytosolic fractions were separately isolated from each group and blotted with anti-NF-κB/p65, anti-GAPDH (cytoplasmic marker), and anti-Lamin B (nuclear marker) antibodies, respectively. **d.** Western blotting was performed with the specified antibodies (related to the region upstream of NF-κB) using lysates from MCF-7 cells exposed to 10 μM BIX for the indicated amounts of time. **e.** The effect of an NF-κB inhibitor, CAPE, on Beclin-1 expression. MCF-7 cells were incubated with 10 mM CAPE combined with or without 10 μM BIX for 4 h. Western blotting was performed with anti-Beclin-1 antibody.

**Figure 4:**
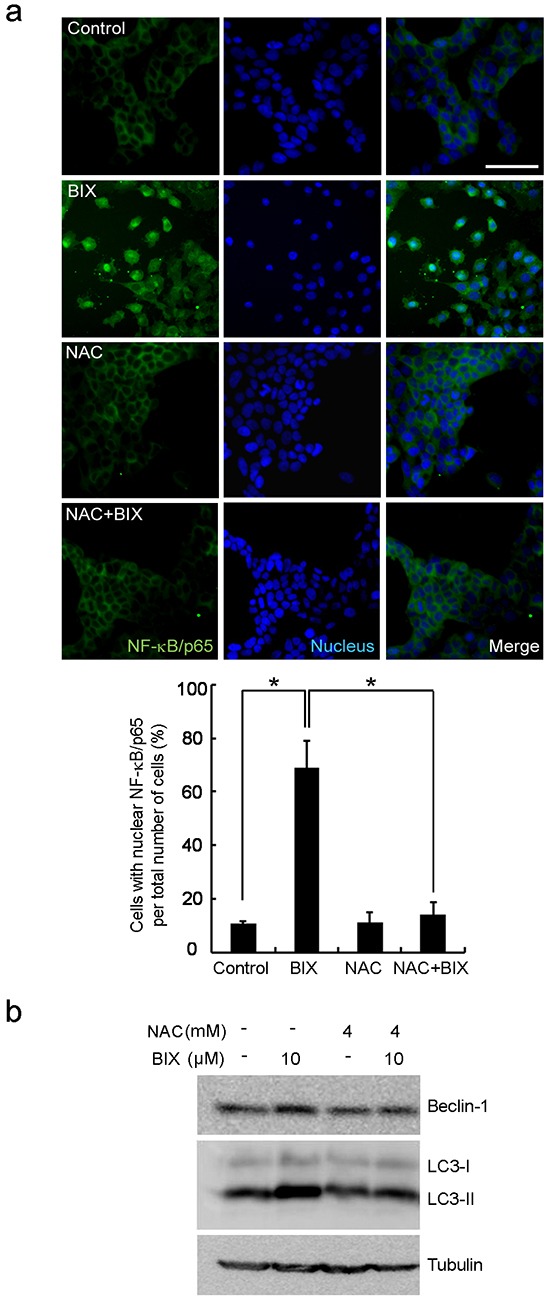
Intracellular ROS-mediated activation of autophagy in response to EHMT2 inhibition **a.** NF-κB nuclear translocation was inhibited by a ROS scavenger, NAC. MCF-7 cells were treated with 10 μM BIX for 4 h in the presence of 4 mM NAC. Cells were then examined by fluorescence microscopy. NF-κB was detected using a Alexa488 (green)-conjugated antibody against the 65 kDa subunit. Nuclei were visualized with Hoechst 33258 (blue). Scale bar, 50 μm. Lower panels show the quantification of translocated NF-κB/p65. The percentage of cells with nuclear NF-κB/p65 is indicated, error bars represent the S.D. (four experiments with at least 50 cells analyzed per condition and experiment). Data are expressed as the means ± SEM of three independent experiments. *P < 0.05 compared with BIX by one-way ANOVA. **b.** Inhibition of ROS by NAC resulted in the reduction of Beclin-1 expression. MCF-7 cells were treated with 10 μM BIX for 4 h in the presence or absence of 4 mM NAC. Western blotting was carried out using the specified antibodies.

As the promoter of *Beclin-1* (analyzed in Figure 2) covers the putative NF-κB binding site (κB sites: GGGACTTTCC) [6, 7], we predicted NF-κB to be a transcription factor responsible for *Beclin-1* transcription. By performing ChIP using antibody against the p65, subunit of NF-κB, we found that p65 was recruited to the promoter of *Beclin-1* by BIX in both MCF-7 and HS578T cells (Figure 3a). Treatment with BIX markedly increased the nuclear translocation of p65 within 4 h (Figure 3b–3c and Supplementary Figure S2a) as well as the phosphorylation of I kappa B kinase α/β (IKKα/β) and NF-κB inhibitor alpha (IκBα) in MCF-7 cells exposed to BIX (Figure 3d). Additionally, treatment with BIX increased the basal levels and phosphorylation of p38 and c-Jun N-terminal kinase (JNK) in MCF-7 cells, which are both associated with NF-κB activation (Supplementary Figure S2b). To better characterize the role of NF-κB in the transcriptional activation of *Beclin-1*, we used an inhibitor of NF-κB, caffeic acid phenethyl ester (CAPE), and a dominant-negative construct of IκBα, pCMV-d/n IκBα. Both CAPE treatment and transfection of pCMV-d/n IκBα resulted in a substantial reduction in the expression of Beclin-1 induced by BIX (Figure 3e and Supplementary Figure S2c). These findings suggested that activation of the IKK/NF-κB pathway enhanced BIX-induced re-expression of Beclin-1. As it has been reported that the BIX-triggered accumulation of intracellular ROS can induce autophagy [31] and that ROS activates NF-κB via the activation of IKK [32], we further tested whether BIX-triggered ROS could activate this process in MCF-7 and HS578T cells. Indeed, pretreatment with N-acetyl-l-cysteine (NAC) effectively blocked the nuclear translocation of p65 (Figure 4a) and the expression of Beclin-1 driven by BIX (Figure 4b and Supplementary Figure S3), indicating that the accumulation of ROS driven by BIX preceded Beclin-1 induction.

### EHMT2 interacts with DNMT1 within the promoter of *Beclin-1* to achieve synergistic transcriptional activation

**Figure 5:**
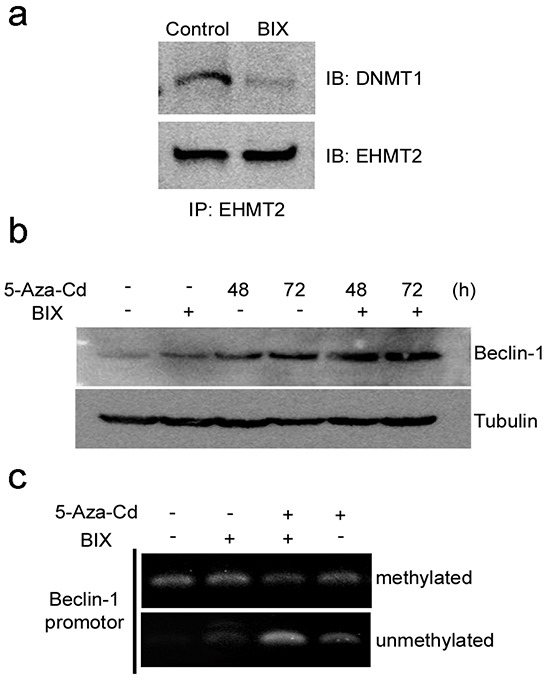
Coordination of DNMT1 and EHMT2 in the suppression of Beclin-1 expression **a.** Western blotting after IP showed that BIX treatment reduced the physical binding between EHMT2 and DNMT1. **b.** Representative Western blots for the detection of Beclin-1 expression before and after MCF7 cells were treated with 10 μM 5-Aza-Cd, 10 μM BIX, or a combination of both treatments. **c.** Methylation-specific PCR (MSP) analysis of DNA methylation in the *Beclin-1* promoter region in MCF7 cells treated with 10 μM 5-Aza-Cd for 48 h, 10 μM BIX for 1 h, or a combination of both treatments.

Recently, it was established that EHMT2 coordinates DNA methylation by DNMT1 [33], eliciting aberrant hypermethylation of DNA and H3K9, which have been linked to the silencing of many critical tumor suppressor genes during neoplastic progression [34, 35]. In accord with these reports, immunoprecipitation with an EHMT2 antibody revealed that exposure to BIX reduced interactions between EHMT2 and DNMT1, which were abundant in untreated MCF-7 cells (Figure 5a). Treatment with an inhibitor of DNMT1, 5-Aza-Cd, re-expressed Beclin-1 and demethylated DNA of the *Beclin-1* promoter in MCF-7 cells (Figure 5b and 5c). Moreover, combined treatment with 5-Aza-Cd and BIX induced greater re-expression of Beclin-1 and demethylation of the promoter compared with the treatment of either BIX or 5-Aza-Cd alone (Figure 5b and 5c). Thus, the combined treatment showed synergistic effects on *Beclin-1* expression.

### Increased expression of EHMT2 shows a positive correlation with suppression of Beclin-1 and a poor prognosis in breast cancer patients

**Figure 6:**
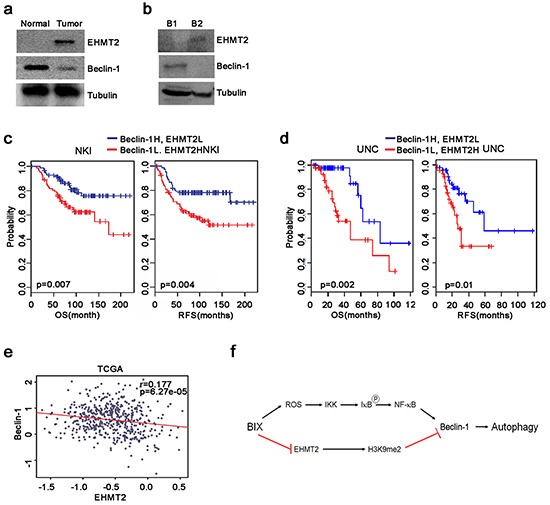
Correlation of high EHMT2 and low Beclin-1 expression levels with a poor prognosis in breast cancer patients **a.** Comparison of the expression of EHMT2 and Beclin-1 in lysates from normal and tumor breast tissue by Western blot. **b.** Western blots of EHMT2 and Beclin-1 in cell lysates from breast cancer patient tumor tissues. **c, d.** Breast cancer patients in the NKI (*n* = 295, left panel) and UNC (*n* = 223, right panel) cohorts were dichotomized based on the expression of Beclin-1 and *EHMT2* and patients with relatively high expression levels of both Beclin-1 and EHMT2 or relatively low expression levels of both Beclin-1 and EHMT2 were identified for analysis. **e.** The correlation between Beclin-1 and EHMT2 expression in TCGA breast cancer patients was estimated using a Pearson's correlation test. **f.** Schematic diagram delineating the reactivation of Beclin-1. Treatment with BIX reduced the levels of H3K9me2, leading to an open chromatin structure. NF-κB can then be recruited to the promoter in ROS-dependent manner, resulting in increased transcription of *Beclin-1* and the activation of autophagy.

To ascertain whether there was a correlation between EHMT2 and Beclin-1 expression in cancer, we examined the expression levels of EHMT2 and Beclin-1 in breast tumor tissue by Western blotting. We observed that EHMT2 was highly expressed in breast tumor tissues (Figure 6a). Remarkably, Beclin-1 showed a completely reciprocal expression pattern compared with EHMT2 in tumor tissue. This observation is consistent with those made using human primary breast and colon cancer cells obtained from patients (Figure 6b and Supplementary Figure S4a). Therefore, we assessed the relationship between the expression of EHMT2 and Beclin-1 and the survival rate of breast cancer patients from two large datasets, the Netherlands Cancer Institute (NKI) and University of North Carolina (UNC) cohorts. In both datasets, patients with high EHMT2 and low Beclin-1 expression exhibited a worse prognosis (Figure 6c and 6d). Furthermore, EHMT2 showed a reciprocal expression pattern with Beclin-1 in The Cancer Genome Atlas (TCGA) of breast cancer patients (Figure 6e). The expression profiles of EHMT2 and Beclin-1 were also inversely correlated with each other in both the NKI and UNC cohorts (Supplementary Figure S4b). Taken together, these findings revealed that increased expression of EHMT2 may contribute to the epigenetic suppression of Beclin-1 expression. The findings reported herein thus provide some new insights into the regulation of Beclin-1 expression in breast cancer.

## DISCUSSION

It is well established that the suppression of Beclin-1 expression results in tumorigenesis via impaired autophagy in cancer. Although the monoallelic deletion of *Beclin-1* has been frequently observed in human breast cancer cell lines and tissues [9], its epigenetic regulation has not yet been elucidated in detail. In this present study, we showed that the expression of ATGs was elevated after the inhibition of EHMT2 by BIX in PCR array analysis, and established that the epigenetic transcriptional activation of *Beclin-1* occurred in breast cancer cells. Treatment of breast cancer cells with BIX removed EHMT2 from the promoter of *Beclin-1,* leading to the reduction of H3K9me2 and resulting in an open chromatin status. These events induced RNA polymerase II to associate with this promoter, thereby initiating transcription (Figure 6f).

Subsequently, we addressed what kind of transcription factor binds to the promoter of *Beclin-1*. Several transcription factors, NF-κB [6, 7], p63 [36], JUN [37], and the forkhead box O (FoxO) transcription factor [38], have been previously implicated in the transcriptional regulation of *Beclin-1*. Among these transcription factors, NF-κB was found to be recruited to the κB site of *Beclin-1* in response to inhibition of EHMT2 in our ChIP experiments. Inhibitory phosphorylation of IκB-α was induced by IKK, which could be activated by BIX-triggered ROS. These findings together suggest that BIX both elevates intracellular ROS and induces the activation of NF-κB resulting in its translocation to the promoter of *Beclin-1*, resulting in transcriptional activation. Autophagy is generally considered to be a cellular survival process that can inhibit protein synthesis and conserve energy under conditions of nutrient deprivation. For this reason, global gene expression appears to be epigenetically suppressed via a reduction in the acetylation of both H4K56 and H4K16 (H4K45ac and H4K16ac) and an increase in the methylation of H3K4 (H3K4me3) during starvation conditions to conserve building blocks and energy. Alternatively, the transcription of genes needed to sustain autophagy must be activated during prolonged starvation. Recently, several reports have shown that the epigenetic modulation of autophagy genes in the nucleus can result in prolonged activation of gene expression [39, 40]. One of these modifications is H3K9me2, which is carried out by the methyltransferase EHMT2 that represses the transcription of *LC3*, *WIPI1*, *DOR*, and *BNIP3* in cancer cells [29]. In this present study, we identified *Beclin-1* as a target gene of EHMT2 and have described a mechanism that promotes its transcriptional activation.

Another regulatory factor that can suppress gene expression is DNMT1, which methylates the CpG islands of many genes. An inhibitor of DNMT1, 5-Aza-Cd, can reduce CpG methylation and reactivate the transcription of genes, such as *p21WAF1*, *CDKN2D*, and *APAF-1* [25, 41, 42]. Moreover, it has been reported that treatment with 5-Aza-Cd reduces both EHMT2 and H3K9me2 in the regulatory regions of silenced tumor suppressor genes, including *p53/p21^Waf1/Cip1^* [43]. In our present study, BIX diminished the interactions of EHMT2 with DNMT1 and the addition of 5-Aza-Cd synergistically reactivated Beclin-1 in breast cancer cells, which could be partially accounted for by the reversal of an ‘epigenetic double lock’ for gene silencing by both DNA and histone methylation. The cross-talk between DNA and histone methylation provides a rationale for using a combination of these epigenetic agents in those breast cancers with silenced tumor suppressor genes.

EHMT2 is overexpressed in many types of cancers and has been suggested to have possible roles in various aspects of tumorigenesis, including cellular differentiation, proliferation, and EMT. Moreover, EHMT2 suppression induces autophagy and represses cell proliferation in neuroblastoma [44, 45] or peritoneal metastasis in ovarian cancer [46]. Although elevated levels of EHMT2 expression have previously been observed in various cancer tissues when compared with normal tissues, the clinical significance of EHMT2 expression in tumors not yet been fully elucidated. We here observed that high expression of EHMT2 was associated with the suppression of Beclin-1 in three independent and publicly available breast cancer datasets—TCGA, UNC, and NKI. Our findings indicated that high EHMT2 and low Beclin-1 expression correlated with reduced overall and recurrence-free survival in patients with breast carcinoma in the UNC and NKI breast cancer datasets. Our findings thus provide preliminary evidence for the role of EHMT2 in the progression of breast cancer via the suppression of *Beclin-1*. Moreover, levels of EHMT2 and Beclin-1 expression may represent useful prognostic markers for patients with breast cancer. Although lower levels of Beclin-1 expression may contribute to the pathogenesis or progression of HER2-enriched basal-like breast cancers with TP53 mutations [47], our current report represents the first study to show a negative correlation of Beclin-1 with EHMT2, which is associated with a poor prognosis in breast cancers.

In conclusion, the epigenetic downregulation of a tumor suppressor gene, *Beclin-1*, by EHMT2, occurs in breast cancer cell lines. Our finding that EHMT2 is overexpressed in breast cancer cells suggests that the development of a novel EHMT2 inhibitor may provide a mechanism for activating an autophagy initiating gene, *Beclin-1,* in cancer cells. Additionally, our study provides a rationale for therapies administered in combination with 5-Aza-Cd to effectively reprogram the transcription of silenced genes in tumor cells based on assessments of the expression levels of EHMT2 and DNMT1.

## MATERIALS AND METHODS

### Cell culture and drug treatments

MCF-7, MCF-7 with GFP-LC3 (GFP-LC3-MCF-7) (1), and HS578T cells were cultured in RPMI1640 (Gibco, 22400-105) containing 10% fetal bovine serum (FBS; Gibco, 16000-044) and 1% penicillin/streptomycin (Invitrogen, 15140-122) at 37°C in a 5% humidified CO_2_ incubator. Primary breast cancer tissues were obtained from consenting patients, and the study protocol was approved by the Research Ethics Board at Asan Medical Center. Tumor specimens were minced with scissors and subsequently digested in Minimum Essential Medium (MEM; Gibco, 11095-098) that contained 1 mg/mL type I collagenase (Sigma, C2799) at 37°C for 2 h. Cells were washed with medium containing 10% FBS, followed by a phosphate-buffered saline (PBS, pH 7.4) wash to remove the FBS. Cells were plated in Mammary Epithelial Basal Medium (MEBM; Lonza, cc-3151) and cultured at 37°C in a 5% CO_2_ incubator. Actinomycin D (ACD; A9415), BIX-01294 (BIX; B9311), caffeic acid phenethyl ester (CAPE; C8221), cyclohexamide (CHX; C1810), 5-Aza-2′-deoxycytidine (5-Aza-Cd; A3656), and N-acetyl-L-cysteine (NAC; A9165) were purchased from Sigma.

### PCR array assay

The Human Autophagy RT2 Profiler PCR Array (SABiosciences, PAHS-084Z) was used to study autophagy-specific gene expression profiles in accordance with the manufacturer's recommendations. Briefly, total RNA was isolated from different experimental groups using Trizol (Invitrogen, 15596-018). Potential genomic DNA contamination was removed from samples by treatment with RNase-free DNase (Invitrogen, 79254) for 15 min at 37°C. The RNA concentration and purity were determined using a NanoDrop ND-1000 (Thermo Scientific). First-strand cDNA was synthesized from 1–2 μg total RNA using SuperScript III Reverse Transcriptase (Invitrogen, 18080044). After cDNA synthesis, real-time PCR was performed using SuperArray PCR master mix (Qiagen, 330503) and a Roche LightCycler® 480 (96-well Block) according to the manufacturer's instructions. Amplification data (fold-changes in Ct values of all genes) were analyzed using the ΔΔCt method.

### RNA extraction and reverse transcription-PCR

Total RNA was prepared using an RNeasy kit (Qiagen, 74104). Complementary DNA was synthesized using an RT^2^ First Strand Kit (Qiagen, 330401) according to the manufacturer's instructions. Polymerase chain reaction was carried out in a C1000™ Thermal Cycler (Bio-Rad) with *Beclin-1* primers (forward 5′-ACT GTG TTG CTG CTC CAT GC-3′ and reverse 5′-CCC AAG CAA GAC CCC ACT TA-3′). After an initial denaturation at 94°C for 20 sec, DNA was amplified using 30 cycles of 94°C for 20 sec, 60°C for 30 sec, and 72°C for 1 min, followed by a final extension step at 72°C for 5 min. Amplification products obtained by PCR were separated on a 1.5% agarose gel.

### Western blot analysis

Cell lysates were prepared for Western blotting as previously described [31]. Immunoblots were probed with the following antibodies: anti-Beclin-1, anti-NF-κB/p65, anti-p-IKKα/β, anti-p-IκB, anti-p38, anti-p-p38, anti-JNK, and anti-Tubulin (Cell Signaling, 3738, 8242, 2697, 2859, 9212, 9211, 9292, 2125), anti-EHMT2 (Millipore, 09-071), anti-LC3 (NOVUS, NB100-2220), anti-H3K9me2 (Abcam, ab1772), and anti-Lamin B (Santa Cruz, sc-6216) followed by incubation with the appropriate secondary antibodies conjugated to horseradish peroxidase (Pierce, 32460). Immobilon™ Western ECL solution (Millipore, WBKLO500) and a Kodak Image Station 4000 MM (Kodak, 745280) were used to visualize immunoreactive bands. Lamin B or tubulin was used as a loading control.

### RNA interference, overexpression of EHMT2, and transfection

Control non-targeting siRNA (sc-37007) and EHMT2 siRNA (sc-43777) were obtained from Santa Cruz. The pcDNA3 (empty vector, MOCK) and pcDNA3-Flag-EHMT2 (FLAG-EHMT2) constructs were obtained from SH Baek (Seoul National University). GFP-LC3-MCF-7 cells were transfected with 0.3 μM siRNA or 1 μg pcDNA-Flag-EHMT2 using Lipofectamine 2000 (Invitrogen, 11668-019), cultured for 2 days, and then used in further experiments.

### Chromatin immunoprecipitation assay

Chromatin immunoprecipitation was performed using an EZ-Magna ChIP™ A/G Kit (Millipore, 17-10086) according to the manufacturer's instructions. Briefly, cells treated with BIX were fixed in 1% formaldehyde and then sonicated. After centrifugation, supernatants containing immunocomplexes were incubated with anti-H3K9me2 (Millipore, 17-648), anti-EHMT2 (Novus, NBL1-10164), anti-RNA pol II (Millipore, 05-623), or anti-NF-κB/p65 (Millipore, 17-10060) at 4°C overnight. Protein G-conjugated magnetic beads were added to lysates and the mixtures were rotated for a further 2 h at 4°C. After immunocomplexes were washed six times with washing buffer, the DNA fragments bound to H3K9me2, EHMT2, RNA polymerase II, or NF-κB/p65 were eluted and the abundance was analyzed using PCR (35 cycles). The primer pair used for *Beclin-1* sequencing was: 5′-CCC GTA TCA TAC CAT TCC TAG-3′ and 5′-GAA ACT CGT GTC CAG TTTCAG-3′.

### Bisulfite treatment and methylation-specific PCR (MSP)

DNA was treated with sodium bisulfite. In brief, 700 ng DNA was denatured in 50 μl 0.2 mM NaOH at 37°C for 20 min; 30 μl freshly prepared 10 mM hydroquinone (Sigma, Heidelberg, Germany) and 520 μl 3 M sodium bisulfite (Sigma) at pH 5 were added. Incubation was carried out under a layer of mineral oil at 55°C for 14 h. DNA was purified using a Gene Clean kit (Qbiogene, Heidelberg, Germany) and eluted with 100 μl water. Finally, DNA was desulfonated by a 20-min treatment in 0.3 M NaOH at 37°C, followed by ethanol precipitation. DNA pellets were resuspended in 1 mM Tris–HCl, pH 8.0. MSP was performed as described previously. Approximately 100 ng bisulfite-modified DNA was amplified with primer pairs that were specific to unmethylated [U primer pair: d(GGTTGGAGT GTAGTGGTATGATTTT) and d(CTAACCAAAATAATT AAATCCCATC)] or methylated DNA sequences [M primer pair: d(GTTGGAGTGTAGTGGTATGATTTC) and d(CTAACCAAAATAATTAAATCCCATC)], respectively. After an initial denaturation step at 95°C for 3 min, DNA was amplified using 33 cycles of 95°C for 30 s, 62°C (U primer pair, M primer pair) for 30 s, and 72°C for 45 s, followed by a final extension at 72°C for 5 min. Sodium bisulfite-treated DNA was PCR-amplified and separated on a 1.5% agarose gel.

### Immunocytochemistry

Cells grown on glass slides were treated as indicated, fixed with 4% paraformaldehyde (Biosesang, P2031) for 10 min, permeabilized with 0.2% Triton X-100 (Sigma, X100) for 15 min, and then blocked with 1% bovine serum albumin for 1 h at room temperature (RT). Cells were probed with rabbit polyclonal anti-NF-κB/p65 (1:75) overnight at 4°C, followed by incubation with goat anti-rabbit AlexaFluor546 antibody (1:100; Molecular Probes, A-11040) for 1 h at room temperature. After washing, cells were mounted with Fluorescent Mounting Medium (Dako, S3023) and analyzed using fluorescence microscopy. Nuclei were counterstained with 2.5 μg/ml Hoechst 33342 at 37°C for 10 min.

### Subcellular fractionation

Cells were harvested and washed with PBS. Cytoplasmic and nuclear fractions were extracted using a NE-PER Nuclear and Cytoplasmic Extraction reagent kit (Thermo Scientific, 78833) according to the manufacturer's instructions.

### Immunohistochemistry

Paraffin-embedded sections were cleared using boric acid solution by microwave boiling for 15 min to retrieve antigens. Sections were blocked with 3% H_2_O_2_ in methanol for 10 min, washed, and then blocked with endogenous peroxidase. After washing, sections were blocked with 3% bovine serum albumin for 1 h at RT. Staining was performed at 4°C overnight with the indicated antibodies along with anti-EHMT2 (R&D Systems, 1:100), followed by incubation with biotinylated secondary antibodies (BioGenex) for 40 min at RT. Finally, sections were incubated with Strep-ABC complex (Dako, K-0377) for 30 min at RT. Sections were developed using a AEC substrate kit (Vector lab, SK-4200) for 20 min at RT, counterstained with hematoxylin, dried, and mounted using a DAKO aqueous mount (Dako, 003181).

### Gene expression data analysis

Gene expression data are publically available from the National Center for Biotechnology Information Gene Expression Omnibus database (http://www.ncbi.nlm.nih.gov/geo) and The Cancer Genome Atlas data portal site (https://tcga-data.nci.nih.gov/tcga/). All data were processed using BRB (Biometric Research Branch) array tools.

### Statistics

Data were obtained from three or more independent experiments and are presented as means ± SEM. Statistical assessments were performed using one-way ANOVA. Data were considered to be significant at a P value of < 0.05.

## SUPPLEMENTARY FIGURES


