# Correction: Inhibition of EHMT2/G9a epigenetically increases the transcription of *Beclin-1 via* an increase in ROS and activation of NF-κB

**DOI:** 10.18632/oncotarget.27046

**Published:** 2019-07-02

**Authors:** Sang Eun Park, Hye Jin Yi, Nayoung Suh, Yun-Yong Park, Jae-Young Koh, Seong-Yun Jeong, Dong-Hyung Cho, Choung-Soo Kim, Jung Jin Hwang

**Affiliations:** ^1^ Institute for Innovative Cancer Research, Asan Medical Center, Seoul, Korea; ^2^ Asan Institute for Life Sciences, Asan Medical Center, Seoul, Korea; ^3^ Department of Neurology, Asan Medical Center, Seoul, Korea; ^4^ Department of Urology, Asan Medical Center, Seoul, Korea; ^5^ Department of Convergence Medicine, University of Ulsan, College of Medicine, Seoul, Korea; ^6^ Neural Injury Research Lab, University of Ulsan, College of Medicine, Seoul, Korea; ^7^ Department of Urology, University of Ulsan, College of Medicine, Seoul, Korea; ^8^ Graduate School of East-West Medical Science, Kyung Hee University, Yongin, Korea; ^9^ Department of Medicine Engineering, Soon Chun Hyang University, College of Medical Sciences, Asan, Korea

**This article has been corrected:** Due to errors during data processing, partial duplication occurred between Figures 2c, 2e and 4b. In addition, typing errors were made within Figure 6e and Supplemental 4a. In Figure 6e, the correlation coefficient (r value) in the picture is -0.177. In the original picture, the ‘-’ is missing. In Supplementary figure 4a, the labeling of BECN-1 and EHMT2 were reversed. The authors declare that these corrections do not change the results or conclusions of this paper.

Original article: Oncotarget. 2016; 7:39796–39808 . https://doi.org/10.18632/oncotarget.9290

**Figure 2:**
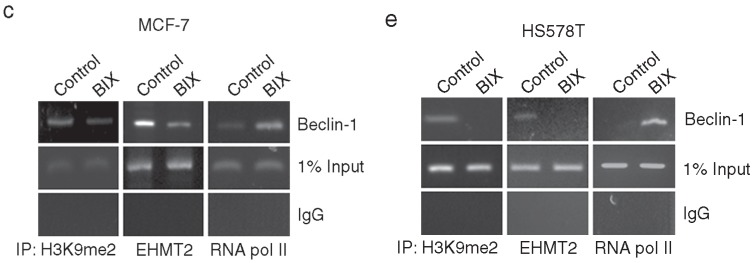
Epigenetic transcriptional activation of *Beclin-1* by EHMT2 inhibition. **c**, **d** and **e**. MCF-7 cells and HS578T cells were treated with 10 μM BIX for 4 h or MCF-7 cells were transfected with 1 μM siEHMT2. Treated cells were analyzed by ChIP. The ChIP analysis performed with anti-H3K9me2, anti-EHMT2, and anti-RNA pol II antibodies was compared with normal rabbit IgG as a negative control. An equal amount (input) of DNA–protein complex was applied. Real-time quantification of the *Beclin-1 *promoter sequences was carried out with anti-H3K9me2 ChIP, anti-EHMT2 ChIP, and anti-RNA pol II ChIP (right panel). Results are presented relative to the input and are the fold-changes over the control expressed as means ± SEM of three independent experiments. *P < 0.001 compared with control or siCont by one-way ANOVA.

**Figure 4:**
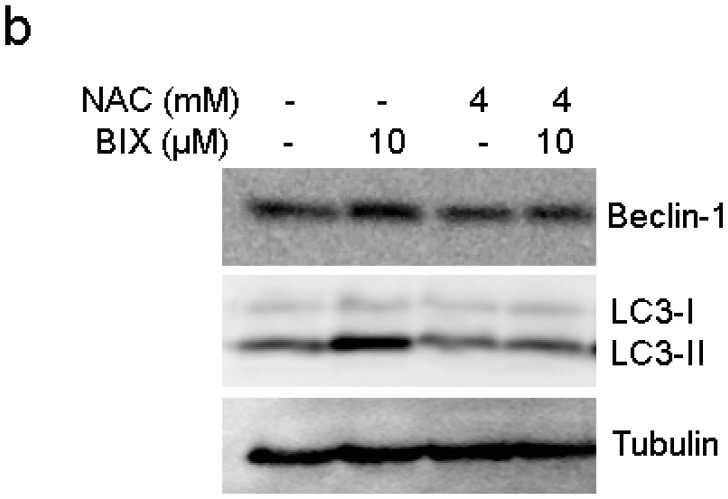
Intracellular ROS-mediated activation of autophagy in response to EHMT2 inhibition. **b.** Inhibition of ROS by NAC resulted in the reduction of Beclin-1 expression. MCF-7 cells were treated with 10 μM BIX for 4 h in the presence or absence of 4 mM NAC. Western blotting was carried out using the specified antibodies.

