# Supplementary material for: Inhibition of EHMT2/G9a epigenetically increases the transcription of Beclin-1 via an increase in ROS and activation of NF-κB
Source: Oncotarget. 2016 May 11;7(26):39796–808. doi: 10.18632/oncotarget.9290 (PMC5129971; doi:10.18632/oncotarget.9290)
Supplement: Supplementary file 1 [file oncotarget-07-39796-s001.pdf]

SUPPLEMENTARY FIGURES

a Genes up- and down-regulated in BIX 4h treated

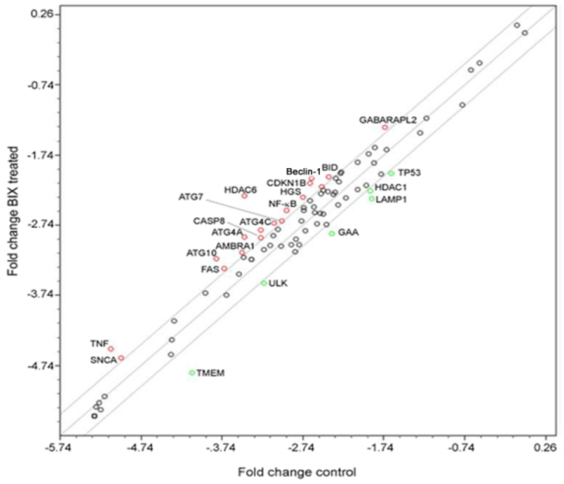

b

Genes up-regulated or down-regulated in BIX 4h treated

Gene Symbol	Fold Regulation
HDAC6	13.6737
TNF	4.0652
ATG10	3.8727
Beclin-1	3.6469
ATG4A	3.5884
CDKN1B	3.2415
ATG4C	2.7895
NFKB1	2.5432
HGS	2.4909
ATG7	2.4116
GABARAPL2	2.395
BID	2.308
AMBRA1	2.308
SNCA	2.2553
FAS	2.2501
CASP8	2.2038
ATG12	2.0658
BAD	2.0658

Gene Symbol	Fold Regulation
TMEM74	-5.3889
GAA	-3.0667
LAMP1	-2.9417
TP53	-2.2815
HDAC1	-2.2346
ULK2	-2.1936

Genes up-regulated in BIX 24h treated

Gene Symbol	Fold Regulation
TNF	65.5426
ATG4B	11.1659
WIPI1	3.2967
Beclin-1	3.2363
GABARLAPL1	3.0831
MAP1LC3A	3.0688
MAP1LC3B	2.8832
BNIP3	2.8436
NFKB1	2.684
SNCA	2.2569
ATG4A	2.0294

Supplementary Figure S1: RT2 Profiler PCR array analysis for autophagy related gene expression in BIX-treated MCF7 cells. a. and **b.** The expression of 84 key genes involved in autophagy were compared between control and cells without or with 10 μM BIX for 4, 24 h. Graph representing fold change in the level of mRNA in cells treated with BIX against untreated cells. Each circle represents an autophagy gene; those with largest fold-changes are indicated in red. The central line represents no changes in expression; above the central line, genes whose expression is increased: below, those with reduced levels. Grey lines indicate 2-fold increase or decrease.

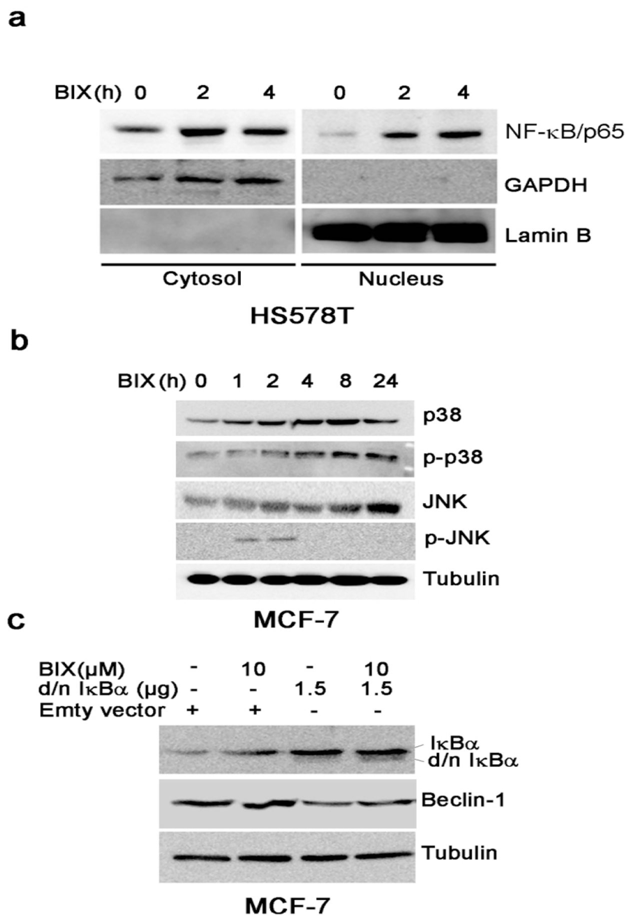

Supplementary Figure S2: a. BIX-induced NF-κB nuclear translocation. HS-578T cells were treated with 10 μM BIX-01294 (BIX) for indicated times. The nuclear and cytosolic fractions were separately isolated from each group and blotted with antibodies to NF-κB/65, GAPDH (cytoplasmic marker) and Lamin B (nuclear marker), respectively. **b.** Western blot analysis was performed with the antibodies specified (associated with activation of NF-κB) using the lysates from MCF-7 cells exposed to 10 μM BIX for indicated times. **c.** Inhibition of NF-κB activation and NF-κB-mediated Beclin-1 expression in MCF-7 cells by transfected d/n IκBα (IκBα dominant/negative vector). MCF-7 cells transfected with an empty vector (lane 1,2), and MCF-7 stably transfected with d/n IκBα (lanes 3,4).

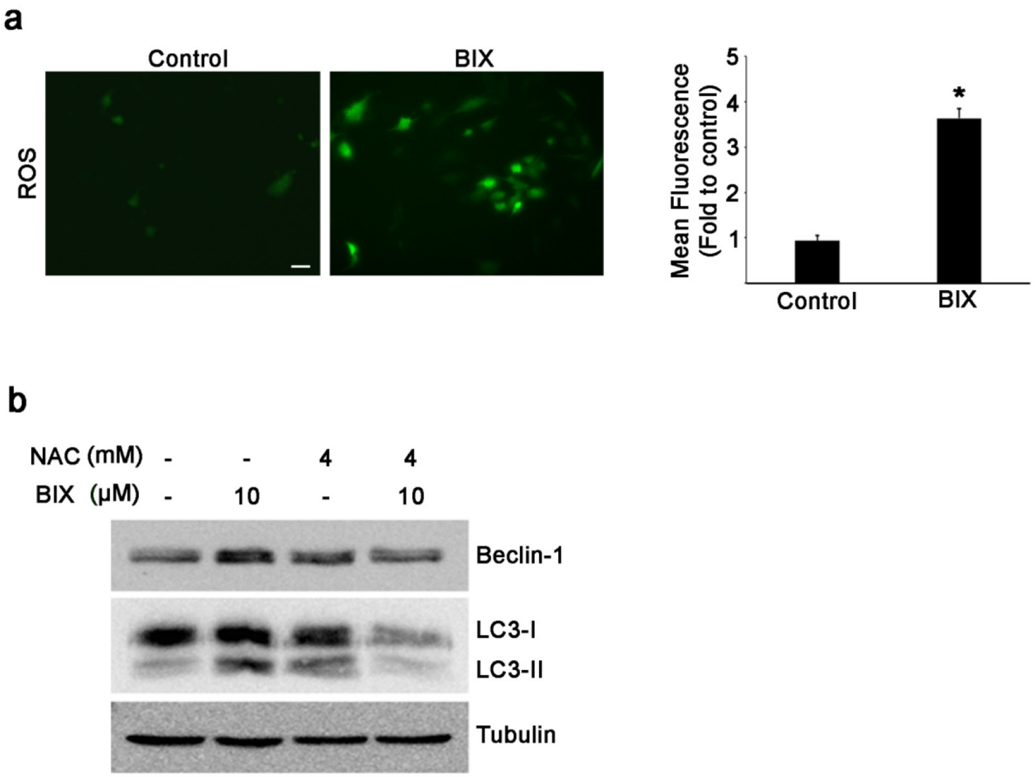

Supplementary Figure S3: BIX induces intracellular ROS-mediated autophagy in HS578T cells. a. HS578T cells were treated with 10 µM BIX for 48 h, and stained with 1 µM CM-H2DCF-DA for 30 min. The level of ROS was examined using fluorescence microscopy. Scale bar: 50 µm. Bar graph represents fold increase of ROS of three independent experiments expressed as means ± SEM *P < 0.05 by one-way ANOVA. **b.** HS578T cells were treated with 10 µM BIX for 4 h in the presence or absence of 4 mM NAC. Western blot analysis was performed using the specified antibodies.

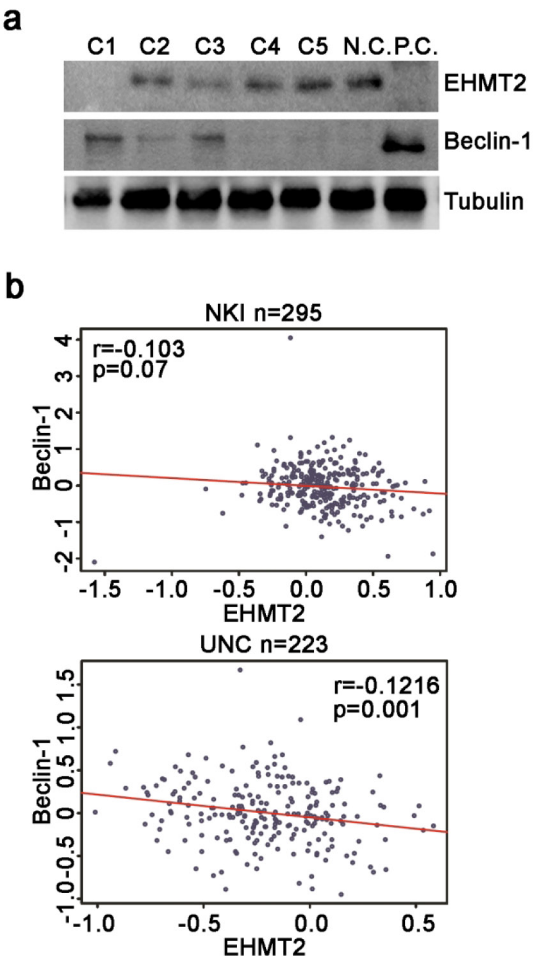

Supplementary Figure S4: Comparison of EHMT2 expression in normal and tumor tissues. a. Comparing the expression of EHMT2 and Beclin-1 in the lysates from tumors of 5 colon cancer patients (C 1–5), and normal colon tissue (N.C.) using Western blot. EHMT2 lysate was used as a EHMT2 positive control (P.C.) **b.** Correlation of Beclin-1 and EHMT2 expression in Netherlands Cancer Institute, NKI (upper box) and University of North Carolina, UNC (lower box) cohort breast cancer patients was estimated by Pearson's correlation test.
